# The effects of acute kidney injury in a multicenter cohort of high-risk surgical patients

**DOI:** 10.1080/0886022X.2021.1977318

**Published:** 2021-09-27

**Authors:** Henrique Tadashi Katayama, Brenno Cardoso Gomes, Suzana Margareth Ajeje Lobo, Renato Carneiro de Freitas Chaves, Thiago Domingos Corrêa, Murillo Santucci Cesar Assunção, Ary Serpa Neto, Luiz Marcelo Sá Malbouisson, João Manoel Silva-Jr, for the BraSIS study group Investigators

**Affiliations:** a Faculdade de Medicina, Hospital das Clínicas, Universidade de São Paulo, São Paulo, Brazil; b Universidade Federal do Paraná, Curitiba, Brazil; c Faculdade de Medicina de São José do Rio Preto, Hospital de Base, São José do Rio Preto, Brazil; d Hospital Israelita Albert Einstein, São Paulo, Brazil

**Keywords:** Acute kidney injury, surgery, high risk, intensive care unit, risk factors, perioperative

## Abstract

**Background and objectives:**

Patients who develop post-operative acute kidney injury (AKI) have a poor prognosis, especially when undergoing high-risk surgery. Therefore, the objective of this study was to evaluate the outcome of patients with AKI acquired after non-cardiac surgery and the possible risk factors for this complication.

**Methods:**

A multicenter, prospective cohort study with patients admitted to intensive care units (ICUs) after non-cardiac surgery was conducted to assess whether they developed AKI. The patients who developed AKI were then compared to non-AKI patients.

**Results:**

A total of 29 ICUs participated, of which 904 high-risk surgical patients were involved in the study. The occurrence of AKI in the post-operative period was 15.8%, and the mortality rate of post-operative AKI patients at 28 days was 27.6%. AKI was strongly associated with 28-day mortality (OR = 2.91; 95% CI 1.51–5.62; *p* = 0.001), and a higher length of ICU and hospital stay (*p* < 0.001). Independent factors for the risk of developing AKI were pre-operative anemia (OR = 7.01; 95% CI 1.69–29.07), elective surgery (OR = 0.45; 95% CI 0.21–0.97), SAPS 3 (OR = 1.04; 95% CI 1.02–1.06), post-operative vasopressor use (OR = 2.47; 95% CI 1.34–4.55), post-operative infection (OR = 8.82; 95% CI 2.43–32.05) and the need for reoperation (OR= 7.15; 95% CI 2.58–19.79).

**Conclusion:**

AKI was associated with the risk of death in surgical patients and those with anemia before surgery, who had a higher SAPS 3, needed a post-operative vasopressor, or had a post-operative infection or needed reoperation were more likely to develop AKI post-operatively.

## Background

Acute kidney injury (AKI) is characterized by a rapid and significant decrease in the glomerular filtration rate (GFR) and is usually of multifactorial origin [1]. Studies that have evaluated perioperative AKI were performed mostly after cardiac and vascular surgery, and there are still major deficiencies in the literature regarding the development of AKI in patients undergoing non-cardiac surgery because most recent studies involve retrospective analyses [2,3].

The development of AKI is commonly associated with sepsis, low cardiac output, and the post-operative period of major surgeries [4]. One out of every three cases of AKI occurs during the perioperative period [5]; such cases represent ∼18–47% of the cases of hospital-acquired AKI [6].

The incidence of AKI in surgical patients varies according to the type and severity of the surgery, with reported rates of 19% after cardiac surgery and ∼12–13% after general and thoracic surgery [7]. In a large epidemiological study of patients undergoing major non-cardiac surgery in intensive care units (ICUs) in Brazil, 30% of patients had post-operative complications, and AKI was the post-operative complication with the second-highest occurrence rate. Post-operative complications are common in high-risk patients after major surgery [8].

Therefore, we hypothesize that perioperative AKI is common and is associated with a worse prognosis. Since there are few prospective data on post-operative surgical patients in intensive care, assessing the incidence and characteristics of patients undergoing major non-cardiac surgery to develop post-operative AKI is relevant to adequately improve the therapeutic management of these patients.

The main objective of this study was to evaluate the incidence, impact on outcomes and main risk factors for developing AKI in patients who underwent non-cardiac surgery who developed AKI after admission to the ICU. Because of the importance of fluid balance in surgical patients, we also evaluated the association between this variable and the development of AKI.

## Methods

This is a pre-planned secondary analysis of a multicenter study recently published [8]. Then, a prospective, multicenter cohort study was conducted between 1 May and 1 November 2018, with a 28-day follow-up period. This study was approved by the Research Ethics Committee of the study coordinating center, Hospital Israelita Albert Einstein (CAAE: 55828016.1.1001.0071), and of all participating centers. A signed written informed consent form was obtained from all patients or their respective legal representatives. Two of the participating centers were exempted from providing the form due to the observational nature of the study.

All patients in the BRASIS study [8] for whom did not have AKI before surgery were selected for this secondary analysis. Patients aged 18 years or older who were undergoing non-cardiac surgery requiring post-operative ICU care were included. Because the criteria for determining the need for post-operative intensive care were not standardized among the centers, all patients with this indication were considered high-risk.

Patients with AKI before surgery, chronic kidney disease, terminal cancer, those receiving palliative care, and those with severe liver failure (Child C) were excluded due to lower, or no prospect of cure, thus their inclusion could lead to inaccurate results. Pregnant women were also excluded. Furthermore, we excluded patients with a length of hospital stay of <12 h because it was not possible to determine whether they were not considered high-risk and for not being able to have follow-up information for at least two days in the ICU. Patients who were readmitted to the ICU during the same hospitalization were excluded to avoid repeating them in the study more than once.

The following variables were evaluated: age; type of surgery; American Society of Anesthesiologists (ASA) classification; Simplified Acute Physiology Score III score (SAPS 3); Sequential Organ Failure Assessment score (SOFA); previous comorbidities; the presence of infection, sepsis or septic shock during ICU stay; creatinine level (mg/dl) at admission and during ICU stay; daily fluid balance; mean arterial blood pressure at ICU admission; mechanical ventilation; vasoactive drugs; length of hospital stay and complications. Day 1 was considered the day of surgery plus admission to the ICU before 11:59 pm. Baseline serum creatinine was the pre-operative value.

Then, AKI was identified by the presence of at least one of the following parameters, Kidney Disease Improving Global Outcomes (KDIGO) criteria 2012 diagnostic score: increase in SCr by ≥0.3 mg/dl (≥26.5 µmol/l) within 48 h; increase in SCr to ≥1.5 times baseline pre-operative, which is known or presumed to have occurred within the prior 7 days; urine volume <0.5 mL/kg/h for 6 h or need for renal replacement therapy during ICU stay in patients with no history of chronic kidney failure [9,10]. Creatinine was measured at the entrance and reassessed in the first 5 days of ICU stay. Oliguria was assessed for 24 h to classify AKI severity.

The fluid balance was calculated as the difference between the infused fluids (crystalloids, colloids, fluid-diluting drugs, blood derivatives, and fluid *via* nasogastric tube) and eliminated fluids (*via* diuresis, bleeding, dialysis, and drains). The need for transfusion was appointed when hemoglobin values were below 8 g/dl intraoperatively. Arterial hypotension was defined when mean blood pressure values intra- and post-operative presented a 30% drop from baseline preoperative values for at least 30 min.

The other post-operative complications were defined as follows: cardiovascular, based on the need for vasopressors for more than 1 h despite adequate volume resuscitation; respiratory, in the presence of a partial pressure of arterial oxygen to fraction of inspired oxygen ratio (PaO_2_/FiO_2_) <200 in patients without previous lung disease, the need for reintubation or the failure to wean from mechanical ventilation during the post-operative period; neurological, based on a sharply fluctuating and non-zero Richmond Agitation-Sedation Scale (RASS) score [11], within 24 h and agitation (determined by a RASS score ≥ +2); and gastrointestinal, in the presence of acute abdominal distension, uncontrolled nausea or vomiting, or moderate- to high-output fistulas.

The main outcome was 28-day mortality after surgery, which was evaluated face-to-face or by telephone. A 28-day follow-up period was chosen to standardize the follow-up time specifically related to surgery.

### Statistical analysis

Considering data from the literature, we assumed a renal complication rate of 15% in high-risk surgical patients [4,7,12]. Therefore, we estimated that at least 1000 patients would be required to conduct the study with the inclusion of fifteen explanatory variables in a robust logistic regression model, with 28-day mortality as the dependent variable. Fortunately, this result was the same found in the first study [8], the current study just did not consider AKI patients before surgery.

Categorical variables are presented as absolute and relative frequencies. Quantitative variables are expressed as the mean and standard deviation (SD) or as the median and interquartile range (IQR), when appropriate. We used the Kolmogorov–Smirnov test to evaluate the distribution pattern of continuous numerical variables.

Proportions were compared using the chi-square test or Fisher’s exact test, as appropriate. Quantitative variables with multiple measurements were compared using general linear model (GLM) analysis, the consistency of the model was tested using Mauchly's sphericity test, and a *post-hoc* Bonferroni correction was performed in these analyses. The Bonferroni correction set the significance cutoff *p*-value; it was used to detect the time points at which the differences were significant in multiple comparisons.

The associations between explanatory and response variables were evaluated using fixed logistic regression models. Variables that were statistically significant in the univariate analyses (*p* < 0.05) were selected for inclusion in the multiple logistic regression models. Collinearity was first evaluated by examining the dispersion matrix and the Pearson correlation coefficient for continuous variables or the cross-tabulation for categorical variables. We also evaluated collinearity using the variance inflation factor (VIF). Variables with substantial collinearity (VIF ≥ 10) were excluded from the final model. The results of the logistic regression analyses are expressed as the odds ratio (OR) and respective 95% confidence interval (95% CI).

All probabilities of significance (*p*-values) were two-tailed. *p*-Values were considered statistically significant when *p* < 0.05. The Statistical Package for Social Sciences v. 26.0 (SPSS Inc.^®^; Chicago, IL, USA) and R v. 3.4.1 (R Foundation for Statistical Computing, Vienna, Austria) were used to perform the analyses.

## Results

**Figure 1.**
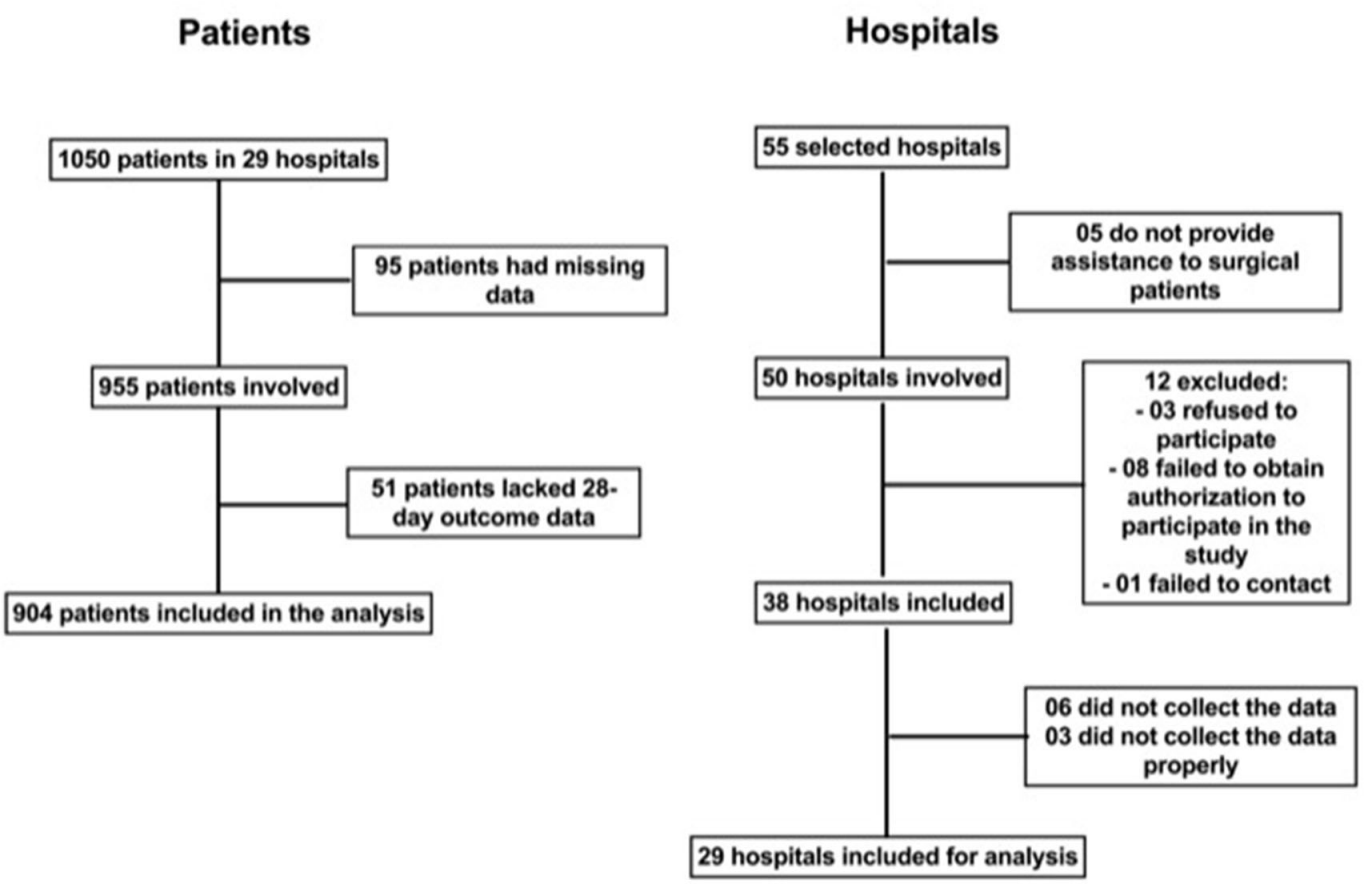
Study flow. Flowchart of study participants.

A total of 55 ICUs from 55 hospitals were selected for participation in the study (chosen for having accepted to participate in the screening). There were no significant differences in operational characteristics among the ICUs when the regions of the country were compared. Twelve of these ICUs (21.8%) were not eligible for participation for different reasons, i.e., 5 ICUs (9.1%) refused to participate because they did not treat the anticipated number of surgical patients, and 9 ICUs (16.4%) returned questionnaires with incomplete study data. Therefore, 29 ICUs participated in the study. During the study period, 25 500 patients underwent non-cardiac surgery. Of these, 904 (3.5%; 95% CI 3.3–3.8%) were admitted to the ICUs and involved in analyze of the study. The patients who entered the study were specifically high-risk surgical patients admitted to the ICU and did not have exclusion criteria (Figure 1).

**Figure 2.**
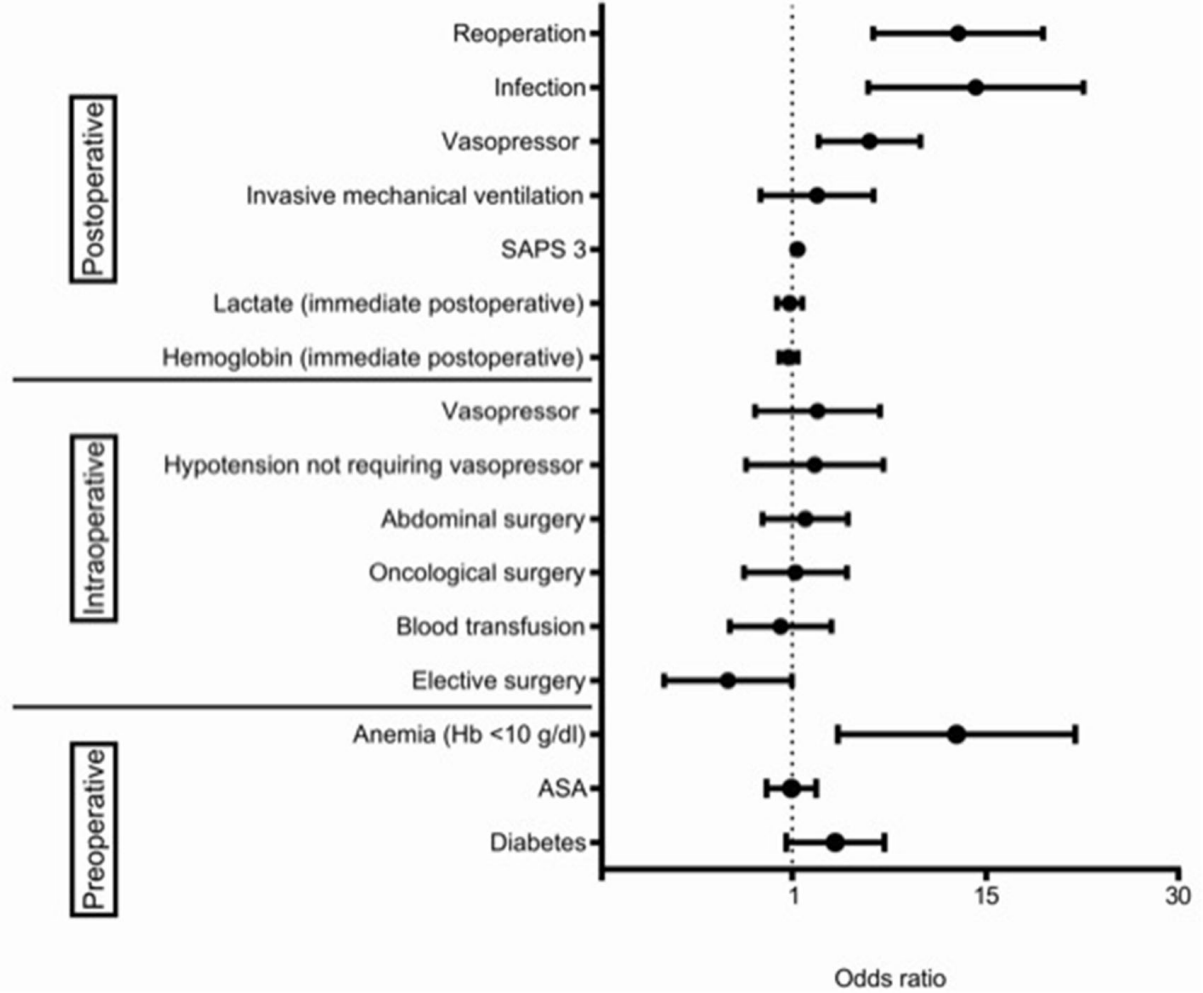
Risk factors related to AKI during the post-operative period (multivariate analysis). ASA: American Society of Anesthesiologists; Hb: hemoglobin; SAPS 3: Simplified Acute Physiology Score III. Area under the curve: 0.844; 95% confidence interval 0.814–0.871 .

The median (IQR) patient age was 62 (50–72) years, and 53.8% of the patients were male. The median (IQR) SAPS 3 was 42 (32–53) points. Approximately 80.4% of the patients had at least one comorbidity, and hypertension, cancer, and smoking were the most frequently occurring.

The median (IQR) length of ICU stay was 2 days (1–4). The median (IQR) length of hospital stay was 9.5 days (5.4–18.6).

The post-operative 28-day mortality rate for the entire cohort was 9.6%, and half of these patients (5.2%) presented with AKI. The total incidence of post-operative complications was 29.9%, with 15.8% of renal complications.

In the logistic regression model, the independent factors associated with 28-day mortality were age (OR = 1.034; 95% CI 1.013–1.054), SAPS 3 (OR = 1.036; 95% CI 1.012–1.061), SOFA at ICU admission (OR = 1.159; 95% CI 1.053–1.275), emergency surgery (OR = 2.7441; 95% CI, 1.197–6.273) and post-operative development of AKI (OR = 2.911; 95% CI 1.507–5.623) (Table 1).

**Table 1.** Factors related to post-operative 28-day mortality (univariate and multivariable analysis).

	Univariate			Multivariate		
	OR	95% CI	*p*-Value	OR	95% CI	*p*-Value
Male sex	1.105	0.658–1.855	0.707			
Caucasian ethnicity	0.926	0.115–7.492	0.943			
Age (per year)	1.019	1.003–1.035	0.017	1.034	1.013–1.054	0.001
BMI (per kg/m^2^)	0.971	0.918–1.027	0.301			
SAPS 3 (per unit)	1.076	1.057–1.096	0.000	1.036	1.012–1.061	0.003
SOFA score at admission (per unit)	1.281	1.198–1.369	0.000	1.159	1.053–1.275	0.002
ASA (per unit)	2.326	1.684–3.213	0.000	1.280	0.884–1.853	0.192
Duration of surgery (per minute)	0.998	0.996–1.000	0.065			
Type of surgery						
Elective	Reference					
Urgent	3.577	1.880–6.806	0.000	1.515	0.677–3.392	0.312
Emergency	6.739	3.659–12.411	0.000	2.741	1.197–6.273	0.017
Surgery						
Head and neck	4.247	1.077–16.754	0.039	3.039	0.901–10.248	0.073
Abdominal	3.988	1.613–9.856	0.003	1.376	0.654–2.894	0.401
Oncological	0.39	0.434–1.622	0.602			
Neurological	3.754	1.331–10.593	0.012	1.340	0.604–2.972	0.472
Orthopedic	2.186	0.774–6.75	0.140			
Vascular	2.124	0.604–7.463	0.240			
Thoracic	1.176	0.195–7.101	0.854			
Urological	2.374	0.680–8.293	0.175			
Gynecological	1.325	0.151–11.596	0.799			
Chronic diseases						
Hypertension	1.083	0.775–1.514	0.639			
Cancer	1.089	0.597–1.920	0.774			
Diabetes	1.199	0.639–2.164	0.557			
Smoking	1.165	0.576–2.221	0.654			
Coronary insufficiency	1.010	0.365–2.393	0.983			
Stroke	2.689	0.689–9.162	0.123			
Chronic obstructive pulmonary disease	1.325	0.471–3.227	0.560			
Alcohol use disorder	1.361	0.435–3.579	0.558			
Arrhythmia	3.007	1.181–7.656	0.021	0.653	0.174–2.453	0.528
Anemia prior to surgery (H*b* < 10 g/dL)	1.211	0.246–5.954	0.814			
Other	1.271	0.738–2.189	0.388			
Type of anesthesia						
General anesthesia	Reference					
Neuraxial anesthesia	0.522	0.183–1.489	0.224			
Combined anesthesia (general + neuraxial)	0.544	0.252–1.172	0.120			
Post-operative acute kidney injury	6.718	3.978–11.343	0.000	2.911	1.507–5.623	0.001

ASA: American Society of Anesthesiologists; BMI: body mass index; 95% CI: 95% confidence interval; Hb: hemoglobin level; OR: odds ratio; SAPS 3: Simplified Acute Physiology Score III; SOFA: Sequential Organ Failure Assessment score.

Area under the curve: 0.843; 95% confidence interval 0.813–0.870.

As shown in Table 1 there was a negative impact of AKI post-operatively. Therefore, this specific population was explored. So, when comparing patients with and without AKI, it was identified that Male sex, higher SAPS 3 and SOFA scores at admission, physical status other than ASA 2, diabetes and anemia, i.e., hemoglobin level before surgery ˂10 g/dl, had a significant association with AKI (Table 2).

**Table 2.** Characteristics of patients presenting with and without acute kidney injury (AKI).

Variable	All patients (*n* = 904)	No AKI (*n* = 761)	AKI (*n* = 143)	*p*-Value
Age (years), mean **±** *SD*	60.1 ± 17.7	59.9 ± 17.5	61.0 ± 18.5	0.489
Median (IQR)	62.0 (50.0–72.0)	62.0 (50.0–72.0)	63.0 (53.0–76.0)	
Male sex	444 (53.8)	358 (51.9)	86 (63.7)	0.012
BMI (kg/m^2^), mean ± *SD*	25.3 ± 4.7	25.3 ± 4.7	25.1 ± 4.8	0.628
Median (IQR)	25.0 (22.0–28.0)	25.0 (22.0–28.0)	25.0 (22.0–27.0)	
SAPS 3, mean **±** *SD*	43.4 ± 15.5	40.8 ± 13.9	56.4 ± 16.6	<0.001
Median (IQR)	42.0 (32.0–53.0)	39.5 (31.0–49.0)	56.0 (45.0–66.7)	
SOFA at admission, mean **±** *SD*	3.16 ± 3.2	2.65 ± 2.74	5.76 ± 4.03	<0.001
Median (IQR)	2.0 (1.0–5.0)	2.0 (1.0–4.0)	5.0 (2.0–8.0)	
ASA 1, *n* (%)	78 (9.2)	58 (8.2)	20 (14.2)	0.438
ASA 2, *n* (%)	405 (47.8)	365 (51.6)	40 (28.4)	0.005
ASA 3, *n* (%)	296 (34.9)	243 (34.4)	53 (37.6)	0.659
ASA 4, *n* (%)	61 (7.2)	38 (5.4)	23 (16.3)	0.163
Chronic diseases, *n* (%)				
None	172 (19.6)	140 (19.0)	32 (22.5)	0.330
Hypertension	396 (44.2)	325 (43.1)	71 (50.0)	0.129
Cancer	191 (21.3)	165 (21.9)	26 (18.3)	0.340
Diabetes	188 (21.0)	149 (19.8)	39 (27.5)	0.039
Smoking	134 (15.0)	108 (14.3)	26 (18.3)	0.222
Coronary insufficiency	67 (7.5)	51 (6.8)	16 (11.3)	0.061
Chronic obstructive pulmonary disease	54 (6.0)	43 (5.7)	11 (7.7)	0.348
Alcohol use disorder	46 (5.1)	34 (4.5)	12 (8.5)	0.051
Arrhythmia	44 (4.9)	34 (4.5)	10 (7.0)	0.200
Stroke	27 (3.0)	21 (2.8)	6 (4.2)	0.357
Anemia prior to surgery (Hb <10 g/dL)	15 (1.7)	8 (1.1)	7 (4.9)	0.001
Intraoperative characteristics of patients				
Time of surgery (min), mean **±** *SD*	282.9 ± 165.0	285.7 ± 164.7	268.5 ± 166.8	0.275
Median (IQR)	240 (180–360)	240 (180–360)	240 (180–360)	
Type of surgery, *n* (%)				<0.001
Elective	613 (69.2)	553 (74.3)	60 (42.3)	
Urgent	147 (16.6)	112 (15.1)	35 (24.6)	
Emergency	126 (14.2)	79 (10.6)	47 (33.1)	
Type of anesthesia, *n* (%)				0.111
General	642 (73.6)	529 (72.4)	113 (80.1)	
Neuraxial	80 (9.2)	68 (9.3)	12 (8.5)	
General and neuraxial	150 (17.2)	134 (18.3)	16 (11.3)	
Diuresis (ml), mean **±** *SD*	816.4 ± 830.8	836.6 ± 813.7	722.2 ± 905.3	0.221
Median (IQR)	500 (300–1007.5)	600 (300–1100)	400 (200–825)	
Fluid balance (mL), mean **±** *SD*	2151.8 ± 1730.4	2113.8 ± 1701.7	2326.6 ± 1853.5	0.208
Median (IQR)	1850 (1000–2925)	600 (300–1100)	400 (200–825)	
Hypotension not requiring vasopressors, *n* (%)	92 (10.5)	55 (7.5)	37 (26.2)	<0.001
Use of vasopressors, *n* (%)	130 (14.9)	83 (11.3)	47 (33.3)	<0.001
Need for blood transfusion, *n* (%)	146 (16.9)	105 (14.5)	41 (29.1)	<0.001
Surgery, *n* (%)				
Oncological	250 (27.9)	222 (29.4)	28 (19.7)	0.018
Neurological	186 (20.8)	160 (21.2)	26 (18.3)	0.433
Head and neck	39 (4.4)	36 (4.8)	3 (2.1)	0.154
Thoracic	53 (5.9)	8 (5.6)	45 (6.0)	0.877
Abdominal	252 (28.1)	193 (25.6)	59 (41.5)	<0.001
Vascular	74 (8.3)	60 (8.0)	14 (9.9)	0.450
Orthopedic	143 (16.0)	121 (16.0)	22 (15.5)	0.868
Urological	48 (5.4)	40 (5.3)	8 (5.6)	0.873
Gynecological	19 (2.1)	18 (2.4)	1 (0.7)	0.202
Other surgeries	56 (6.2)	45 (6.0)	11 (7.1)	0.625
Post-operative characteristics				
MAP (mmHg) at ICU admission, mean **±** *SD*	90.5 ± 18.3	91.0 ± 18.1	87.9 ± 18.8	0.070
Median (IQR)	90.0 (78.7–101.3)	90.3 (79.3–101.8)	88.5 (77.0–100.3)	
Hemoglobin (g/dl) at ICU admission, mean **±** *SD*	11.4 ± 2.2	11.5 ± 2.2	10.8 ± 2.4	0.001
Median (IQR)	11.5 (10.0–12.9)	11.6 (10.1–13.0)	10.8 (9.4–12.4)	
Hematocrit (%) at ICU admission, mean **±** *SD*	33.9 ± 6.9	34.3 ± 6.6	32.1 ± 8.2	0.001
Median (IQR)	34.1 (30.2–38.6)	34.6 (30.8–38.7)	32.9 (27.9–37.0)	
Lactate (mmol/L) at ICU admission, mean **±** *SD*	2.1 ± 1.6	2.0 ± 1.5	2.5 ± 1.9	0.002
Median (IQR)	1.7 (1.1–2.7)	1.7 (1.1–2.6)	1.8 (1.3–3.0)	
Creatinine (mg/dl)				
Day 1, mean **±** *SD*	1.1 ± 1.3	1.0 ± 1.0	1.7 ± 2.0	<0.001
Median (IQR)	0.8 (0.6–1.1)	0.8 (0.6–1.0)	1.0 (0.7–1.6)	
Day 2, mean **±** *SD*	1.1 ± 1.2	0.9 ± 0.9	1.8 ± 2.1	<0.001
Median (IQR)	0.8 (0.6–1.1)	0.8 (0.6–1.0)	1.1 (0.7–2.1)	
Day 3, mean **±** *SD*	1.1 ± 1.2	0.9 ± 0.9	1.7 ± 1.7	<0.001
Median (IQR)	0.8 (0.5–1.2)	0.7 (0.5–1.0)	1.0 (0.6–2.2)	
Diuresis (mL)				
Day 1, mean **±** *SD*	1104.9 ± 1118.5	1152.5 ± 1164.6	853.1 ± 789.6	0.006
Median (IQR)	900 (500–1400)	1150 (700–2000)	1200 (575–1900)	
Day 2, mean **±** *SD*	1246.3 ± 1066.6	1298.4 ± 1105.0	1000.3 ± 821.8	0.002
Median (IQR)	1000 (550–1657)	1050 (600–1700)	812 (350–1500)	
Day 3, mean **±** *SD*	1466.9 ± 1274.2	1444.8 ± 1144.2	1539.7 ± 1634.2	0.468
Median (IQR)	1150 (700–2000)	1150 (700–2000)	1200 (575–1900)	
Invasive MV at ICU admission, *n* (%)	148 (17.7)	81 (11.7)	67 (46.9)	<0.001
Vasopressor use, *n* (%)				
Day 1	157 (18.0)	101 (13.8)	56 (39.4)	<0.001
Day 2	202 (24.0)	123 (17.6)	79 (55.2)	<0.001
Day 3	142 (25.4)	77 (17.8)	65 (51.6)	<0.001
Need for hemodialysis, *n* (%)	18 (2.0)	0 (0.0)	18 (13.4)	<0.001
Duration of MV (days), mean **±** *SD*	5.7 ± 6.3	4.2 ± 5.2	8.3 ± 7.1	<0.001
Median (IQR)	3.0 (1.0–8.0)	2.0 (1.0–5.0)	7.0 (3.0–12.0)	
Length of ICU stay (days), mean **±** *SD*	3.8 ± 5.4	3.1 ± 4.4	8.2 ± 7.9	<0.001
Median (IQR)	2.0 (1.0–4.0)	2.0 (1.0–3.0)	5.9 (2.8–11.0)	
Length of hospital stay (days), mean **±** *SD*	14.4 ± 14.9	13.1 ± 14.2	21.3 ± 17.1	<0.001
Median (IQR)	9.5 (5.3–18.6)	8.5 (4.7–16.6)	16.7 (9.5–29.6)	
Infection, *n* (%)	16 (1.8)	5 (0.7)	11 (7.7)	<0.001
Reoperation, *n* (%)	30 (3.3)	11 (1.4)	19 (13.3)	<0.001
ICU mortality, *n* (%)	41 (4.9)	14 (2.0)	27 (20.3)	<0.001
Hospital mortality, *n* (%)	74 (8.9)	31 (4.5)	43 (31.6)	<0.001
28-day mortality, *n* (%)	68 (9.6)	31 (5.4)	37 (27.6)	<0.001

BMI: body mass index; ICU: intensive care unit; Emergency: surgery required immediately after diagnosis of the problem; Hypotension not requiring vasopressors: reversal of hypotension with volume; MAP: mean arterial pressure; MV: mechanical ventilation; *N*: number of participants; Other surgeries: ophthalmological and plastic surgeries and transplants; SAPS 3: Simplified Acute Physiology Score III; Urgent: surgery required 24–48 h after diagnosis of the problem.

Values are expressed as the mean ± standard deviation, median (IQR-interquartile range), or absolute value (%).

During the intraoperative period, the variables that were significantly associated with AKI were the type of surgery, intraoperative hypotension without the need for reversal with vasopressors (reversal of hypotension with volume), need for vasopressors and blood transfusion, and abdominal and oncological surgery (Table 2).

In addition, during the post-operative period, AKI was more strongly correlated with a lower hemoglobin level, higher arterial lactate level, and mechanical ventilation at ICU admission, in addition to the need for vasopressors during this period, the presence of post-operative infection, and the need for unplanned reoperation (Table 2). It can be noted that from the values of diuresis, creatinine, and need for dialysis that of the AKI patients 13.4% were KIDGO 3, and 86.6% developed KIDGO 1 and 2.

However, when logistic regression was applied to the perioperative variables that were identified as statistically significant in the univariate analysis comparing patients with and without AKI during the post-operative period (except for the male gender and SOFA score variables, because they present collinearity with SAPS 3 score, therefore, these variables did not participate in the logistic regression model), only preoperative anemia (OR = 7.01; 95% CI 1.69–29.07), elective surgery (OR = 0.45; 95% CI 0.21–0.97), SAPS 3 (OR = 1.04; 95% CI 1.02–1.06), post-operative vasopressor use (OR = 2.47; 95% CI 1.34–4.55), post-operative infection (OR = 8.82; 95% CI 2.43–32.05) and the need for reoperation (OR = 7.15; 95% CI 2.58–19.79) were independent factors associated with AKI (Figure 2).

Specifically, regarding daily fluid adjustment during the perioperative period, AKI exhibited an association in the univariate analysis with the total volume and 0.9% saline volume received every 24 h. Nevertheless, when these variables were adjusted for the identified risk factors for developing AKI, there was no association with AKI (Table 3).

**Table 3.** Comparison of patients with or without acute kidney injury (AKI) according to fluids received during the perioperative period, univariate and multivariate analysis (adjusted for SAPS 3, preoperative anemia, elective surgery, post-operative vasopressor use, post-operative infection, and reoperation).

		Univariate			Multivariate	
Variable (daily median)	All patients	No AKI	AKI	*p*-Value	OR (95% CI)	*p*-Value
Total volume (mL)	1900 (1253–2502)	1820 (1250–2500)	2000 (1500–2927)	0.007	1.001 (0.998–1.003)	0.186
mL/kg	26.1 (18.1–37.9)	25.0 (17.6–37.2)	29.3 (21.4–41.2)	0.004		
0.9% saline solution (mL)	1044 (720–2000)	1000 (642–1750)	1500 (1000–2527)	<0.001	1.000 (0.999–1.001)	0.840
mL/kg	16.7 (10.7–27.7)	15.7 (9.9–24.5)	22.0 (14.2–34.1)	<0.001		
Lactated Ringer’s solution (mL)	1500 (1000–2500)	1500 (1000–2500)	1500 (1000–2500)	0.337	1.000 (0.998–1.002)	0.800
mL/kg	23.4 (13.7–38.7)	23.9 (13.9–39.1)	21.5 (13.3–33.6)	0.446		
Plasma-Lyte (mL)	1500 (1000–2164)	1500 (1000–2500)	1150 (500–2000)	0.121	0.999 (0.998–1.007)	0.544
mL/kg	19.5 (12.2–35.7)	19.6 (13.2–39.7)	15.1 (7.8–28.9)	0.127		
Hydroxyethylamide 130/0.4 (mL)	500 (500–1000)	500 (500–500)	1000 (750–1000	0.137	—	
mL/kg	8.9 (7.1–14.1)	8.2 (6.4–11.5)	13.0 (9.7–15.8)	0.152		

OR: odds ratio; 95% CI: 95% confidence interval.

Values are expressed as the median (interquartile range); adjusted OR in the multivariate analysis.

**Figure 3.**
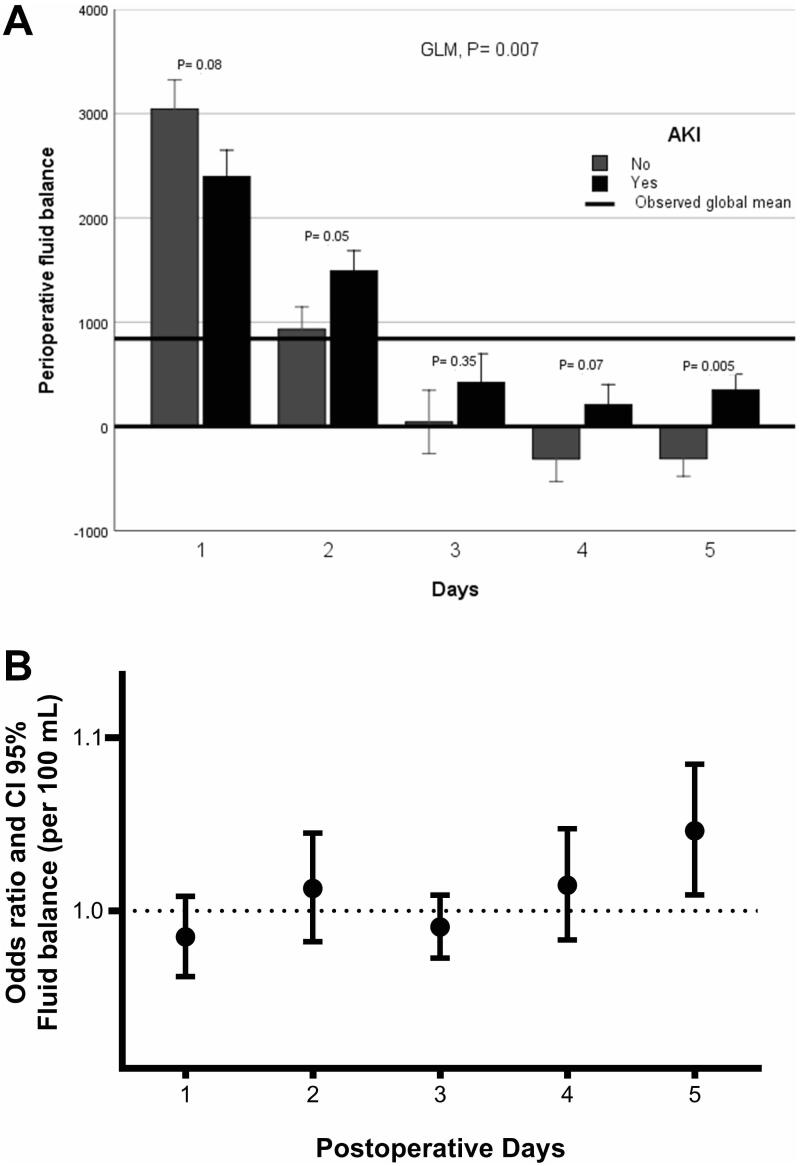
(A) Multiple analysis model (GLM) for variables repeated over time in relation to fluid balance in patients with and without AKI. *p*-Values were corrected by the Bonferroni method and (B) multivariate analysis for daily post-operative fluid balance (per 100 mL) adjusted for SAPS 3, preoperative anemia, elective surgery, post-operative vasopressor use, post-operative infection, and reoperation.

Regarding the evolution of the daily fluid balance in patients with AKI, the GLM showed significant differences (*p* = 0.007) in fluid balance from the day of surgery to the fifth day between patients with and without AKI. The difference was most evident on the second (*p* = 0.05 Bonferroni correction) and fifth (*p* = 0.005 Bonferroni correction) post-operative days. Furthermore, when the daily fluid balance was adjusted for the identified risk factors for developing AKI, only a high fluid balance on the fifth post-operative day was stronger correlated with AKI (OR per 100 mL= 1.051; 95% CI 1.010–1.094) (Figure 3).

## Discussion

The main findings of this study were a high incidence of AKI (15.8%) with high mortality in non-cardiac surgery patients admitted to the ICU and the association of AKI with non-elective surgeries, infection, reoperation, circulatory shock, preoperative anemia, and inappropriate positive fluid balance.

Major surgeries account can reach 40% of cases of hospital-acquired AKI [13]. Patients with AKI admitted to the ICU during the post-operative period in cardiovascular surgery accounted for 7.7–40% of cases [14], and gastrointestinal tract surgery patients accounted for almost 22% [15]. Specific data on surgical patients requiring intensive care are scarce.

It is important to comment that although this study does not include surgical cardiac patients, the findings of our study can be extrapolated in several aspects for these patients, because cardiac surgical patients suffer some complications and have some similar characteristics to high-risk non-cardiac surgery, thus, the risk factors for AKI can be the same [16].

The presence of AKI during the perioperative period in this subgroup of patients undergoing major surgery was associated with unfavorable outcomes, with a hospital mortality rate of 27.6%. In addition, the length of hospital stay of patients with AKI was higher than that of patients non-AKI; patients with AKI spent approximately one extra week in the hospital. Similarly, a large multicenter observational study in European countries reported that mortality among patients with AKI was more than twice that observed in other patients [17]. In a recent study, the occurrence and severity of AKI were strongly associated with the risk of death after surgery. However, the relationship between preoperative renal function, as assessed by serum creatinine-based estimated GFR, and the risk of death depended on patient age and whether AKI developed post-operatively [18].

In the current study, patients who developed AKI were more severe (higher SAPS 3), non-elective operations, had a greater need for advanced support with catecholamines, and frequently had infections.

Non-elective operations make it difficult to prepare and assess the risk for AKI, which prevents the proper management of these patients, making them more vulnerable to this problem [19,20]. In addition, the intensity of the inflammatory response to surgical trauma determines both tissue hypoperfusion and the ischemia-reperfusion response, which intensifies the inflammatory response. Prospective studies have reported a relationship between AKI and sepsis and the need for mechanical ventilation [6,21,22].

There is a close correlation between hypotension and the development of AKI. A recent meta-analysis showed that goal-directed hemodynamic adjustments reduce the likelihood of AKI in orthopedic and abdominal surgery patients [23]. We observed the most frequent use of vasopressors in this group of patients. The duration of intraoperative hypotension (particularly relative to the patient’s normal arterial blood pressure), including brief episodes of mean arterial pressure lower than 55 mmHg, has been associated with kidney injury [5]. Careful maintenance of cardiovascular stability, including fluid infusion, throughout this period, is vital for protecting renal perfusion while avoiding volume overload. A study of high-risk surgical patients showed that the customized maintenance of blood pressure according to the patient's previous levels during surgery was able to reduce organ dysfunction [24]. Moreover, the association between the use of vasoactive drugs and mortality is consistent with the results of other studies [25–27].

Interestingly, a correlation was noted between preoperative anemia (such as hemoglobin concentration <10 g/dl) and impaired renal function. This pathophysiology is multifactorial, but mainly involves a decrease in the oxygen transport capacity and subsequent tissue hypoxia [28]. Patients in the AKI group had more transfusions, which may have induced more systemic inflammation, in addition to having more patients with anemia (Hb < 10 g/dl) at entry, a condition that may have facilitated tissue hypoxia.

Besides, patients who developed AKI had a more positive daily fluid balance for a long time. In Figure 3 it is quite clear that AKI patients maintain a positive balance until the fifth day and non-AKI patients more rapidly decrease the fluid balance.

It is expected that, in many cases, simply restoring the circulating volume does not improve the results and maybe counterproductive [4,5]. Organ edema distorts tissue architecture, impairs oxygen and metabolite diffusion, and obstructs the capillary flow and lymphatic drainage. These effects are particularly pronounced in encapsulated organs, such as the kidney, which cannot accommodate additional volume without significant increases in interstitial pressure and compromised blood flow. Elevated intratubular pressure decreases glomerular filtration and activation of tubuloglomerular feedback, with consequent preglomerular vasoconstriction, which leads to an additional reduction in glomerular filtration [29–31]. Studies have shown that excess fluid is an independent factor for the development of AKI and that in patients with AKI, a more positive fluid balance was correlated with higher mortality [32–35]. Regarding the type of solution used, epidemiologic data suggest that 0.9% saline solution, when compared with balanced salt solutions, such as balanced solutions, may increase the risk of AKI. In addition, there is evidence of harm (increased rates of AKI) with the use of hetastarch solutions, which should generally be avoided [36]. In our study was not detect a negative influence of any type of colloid or crystalloid on the development of AKI.

Based on these, patients should be stratified according to their risk of developing AKI based on their exposure and susceptibility [37,38], and preemptive measures should be taken [5].

## Study limitations

The main strengths of our study are associated with its multicenter nature, as it included ICUs located in several regions of Brazil. However, there was a reasonable rate of refusal to participate in the study that affected external validity to some degree. In addition, the adjusted regression model could be performed with *a priori* defined AKI prediction variables, and no based on statistically important findings from the unadjusted analysis.

There were failures in capturing some relevant data that could have been included in the analyses, such as nephrotoxic agents or humoral factors, contrast exposure perioperatively, and the nature of oncological or vascular surgery. Additionally, the need to obtain informed consent in epidemiological studies, such as this tends to skew the sample due to the non-consenting of more critical patients, whose families may be psychologically fragile. Another aspect to be considered is the lack of standardization among the centers regarding indications for post-operative intensive care and treatments, but this fact does not invalidate the findings, because we tried to show what happens in real practice. Besides, our finding regarding inappropriate positive fluid balance and renal failure only generate a hypothesis, it may not necessarily be a cause-effect relationship. Furthermore, this study was not able to assess other possible causes of renal failures, such as intra-abdominal hypoperfusion and increased airway pressures [39], also long-term complications and mortality, because some complications may have occurred after the study period.

## Conclusion

AKI is a major complication in intensive care surgical patients and is associated with the risk of death, for this reason, it deserves attention in the perioperative. Patients with anemia before surgery, those with a higher SAPS 3, those requiring vasopressors during the post-operative period, and those with post-operative infection or the need for reoperation are the most likely to develop AKI, as are patients with an inappropriate higher perioperative fluid balance for a long time, therefore care should be considered in the perioperative period for these problems. The fluid type did not influence AKI development.

## Data Availability

The datasets used and/or analyzed during the current study are available from the corresponding author on reasonable request.

## Abbreviations

AKI: acute kidney injury
ASA: American Society of Anesthesiologists
GFR: glomerular filtration rate
ICU: intensive therapy unit
KDIGO criteria: Kidney Disease Improving Global Outcomes criteria
RASS: Richmond Agitation-Sedation Scale Score
SAPS 3: Simplified Acute Physiology Score III
SOFA: Sequential Organ Failure Assessment
